# Inhibition of Rac1 in ventral hippocampal excitatory neurons improves social recognition memory and synaptic plasticity

**DOI:** 10.3389/fnagi.2022.914491

**Published:** 2022-07-22

**Authors:** Haiwang Zhang, Youssif Ben Zablah, Haorui Zhang, An Liu, Radu Gugustea, Dongju Lee, Xiao Luo, Yanghong Meng, Song Li, Changxi Zhou, Tao Xin, Zhengping Jia

**Affiliations:** ^1^Department of Neurosurgery, The First Affiliated Hospital of Shandong First Medical University & Shandong Provincial Qianfoshan Hospital, Shandong Medicine and Health Key Laboratory of Neurosurgery, Jinan, China; ^2^Program in Neurosciences and Mental Health, The Hospital for Sick Children, Peter Gilgan Centre for Research and Learning, Toronto, ON, Canada; ^3^Department of Physiology, Temerty Faculty of Medicine, University of Toronto, Toronto, ON, Canada; ^4^The Key Laboratory of Developmental Genes and Human Disease, Ministry of Education, School of Life Sciences and Technology, Southeast University, Nanjing, China; ^5^Department of Neurosurgery, Caoxian People’s Hospital, Caoxian, China; ^6^Department of Geriatrics, The Second Medical Center and National Clinical Research Center for Geriatric Diseases, Beijing, China

**Keywords:** social memory, APP/PS1 mouse model, ventral hippocampus, Rac1, LTP

## Abstract

Rac1 is critically involved in the regulation of the actin cytoskeleton, neuronal structure, synaptic plasticity, and memory. Rac1 overactivation is reported in human patients and animal models of Alzheimer’s disease (AD) and contributes to their spatial memory deficits, but whether Rac1 dysregulation is also important in other forms of memory deficits is unknown. In addition, the cell types and synaptic mechanisms involved remain unclear. In this study, we used local injections of AAV virus containing a dominant-negative (DN) Rac1 under the control of CaMKIIα promoter and found that the reduction of Rac1 hyperactivity in ventral hippocampal excitatory neurons improves social recognition memory in APP/PS1 mice. Expression of DN Rac1 also improves long-term potentiation, a key synaptic mechanism for memory formation. Our results suggest that overactivation of Rac1 in hippocampal excitatory neurons contributes to social memory deficits in APP/PS1 mice and that manipulating Rac1 activity may provide a potential therapeutic strategy to treat social deficits in AD.

## Introduction

Social interaction and memory are essential for our health and success. Alzheimer’s disease (AD), a leading cause of dementia, is a neurodegenerative disease characterized by progressive loss of various forms of memories, including social memory (Filali et al., 2011). Furthermore, impaired social memory in AD patients may lead to patients developing apathy toward social engagement and can result in a preference for introversion. Due to the importance of social interaction for cognition and mental health (reviewed in: Berkman et al., 2000; Leser and Wagner, 2015), social isolation caused by AD could further worsen disease progression (Wilson et al., 2007; Ali et al., 2017). Currently, there is no cure or effective treatment available due to the limited understanding of the pathological mechanisms underlying AD. Numerous studies suggest that the excessive accumulation of amyloid-beta (Aβ) peptides and neurofibrillary tangles in the brain is the most common causative cause of AD (reviewed in: Hardy and Selkoe, 2002; Ballatore et al., 2007). However, other factors such as neuroinflammation, oxidative stress, and injury of cholinergic neurons, may also contribute to the pathogenic process of AD (reviewed in Serrano-Pozo et al., 2011; Spires-Jones and Hyman, 2014; Singh et al., 2016). How Aβ peptides lead to neuronal degeneration and memory loss remain unclear, but evidence has indicated that Aβ accumulation can impair long-term potentiation (LTP) (Walsh et al., 2002; Almeida et al., 2005; Snyder et al., 2005; Hsieh et al., 2006) and promote long-term depression (LTD) (Shankar et al., 2008; Li et al., 2009). LTP and LTD are the most extensively studied forms of synaptic plasticity widely considered to be key mechanisms underlying learning and memory (reviewed in: Bliss and Collingridge, 1993; Kandel et al., 2014). LTP deficits were widely observed in animal models of AD (reviewed in: Palop and Mucke, 2010; Mucke and Selkoe, 2012; Sheng et al., 2012), but further investigations are required to understand the molecular mechanisms by which LTP is affected by the disease and how they are related to memory deficits.

Rac1 is a member of the Rho family small GTPases known to be a central regulator of actin cytoskeleton dynamics, neuronal structures, synaptic plasticity, and memory maintenance (reviewed in: Lamprecht, 2014; Costa et al., 2020; Zhang et al., 2021b). In particular, overactivation of Rac1 has been shown to promote memory decay (Shuai et al., 2010; Gan et al., 2016; Jiang et al., 2016; Liu et al., 2016, 2018). Elevated Rac1 activity was observed in both human AD patients and animal AD models (Mendoza-Naranjo et al., 2007; Borin et al., 2018; Wu et al., 2019). In addition, reducing Rac1 activity improves spatial memory performance in AD (Wu et al., 2019), suggesting that Rac1 overactivation may contribute to spatial memory deficits in AD. However, whether Rac1 dysregulation is involved in other forms of memory impairments associated with AD is unknown. In addition, the brain regions, cell types and underlying mechanisms by which Rac1 leads to memory loss remain unclear. In this study, we suppressed Rac1 activity by viral expression of a dominant-negative (DN) mutant, Rac1-N17, specifically in the excitatory neurons of mouse ventral hippocampus. We showed that reducing Rac1 activity is sufficient to improve social recognition memory and rescue LTP impairment in APP/PS1 (APP) mice. Our results suggest that increased Rac1 activity contributes to social memory deficits in AD and therefore inhibiting Rac1 may provide a potential therapeutic strategy for ameliorating the social behavior deficit observed in AD patients.

## Results

### Impaired long-term potentiation in ventral hippocampus in APP/PS1 mice

**FIGURE 1:**
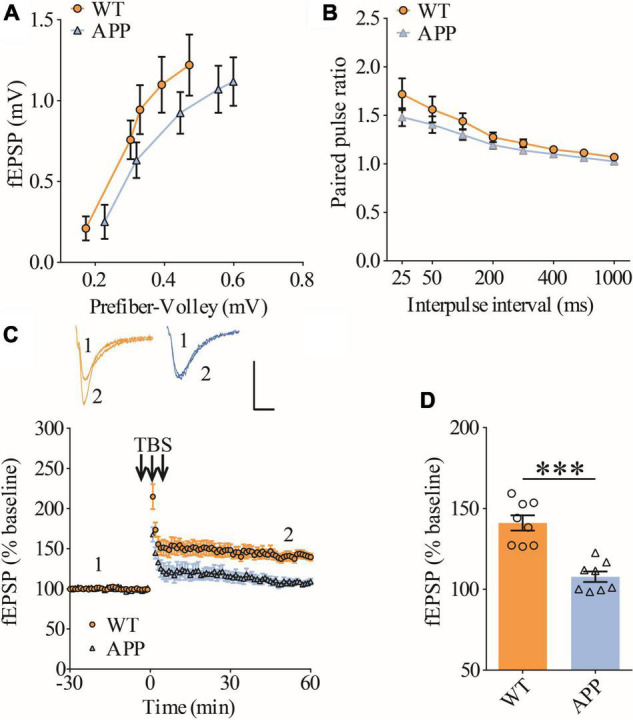
Impaired LTP in ventral hippocampus in APP mice. **(A)** Input output curves of fEPSP showing no differences between WT and APP mice (WT: *n* = 9 slices from 5 mice, APP: *n* = 5 slices from 5 mice; genotype: *F*_(1_,_12)_ = 0.060, *p* = 0.811; prefiber volley: *F*_(4_,_48)_ = 37.27, *p* < 0.001; repeated two-way ANOVA). **(B)** Paired pulse ratio showing no differences between WT and APP mice (WT: *n* = 8 slices from 5 mice, APP: *n* = 7 slices from 5 mice; genotype: *F*_(1_,_13)_ = 1.658, *p* = 0.220; inter-pulse interval: *F*_(7_,_91)_ = 33.74, *p* < 0.001; repeated two-way ANOVA). **(C)** TBS induced LTP at the CA1 synapse in WT and APP mice. Scale bars: 0.4 mV/10 ms. **(D)** Summary graph of last 10 min of recording showing impaired LTP in APP compared to WT mice (WT: *n* = 8 slices from 5 mice, APP: *n* = 8 slices from 5 mice, *p* < 0.001, two-tailed *t*-test). ***P < 0.001.

To confirm synaptic deficits in APP mice, we carried out electrophysiological recordings at the Schaffer collateral-commissural pathway (CA3-CA1 synapse) in the ventral hippocampus, a brain region critically involved in social recognition memory (Okuyama et al., 2016). We first examined basal synaptic transmission using various stimulation intensities (1, 2, 3, 4, and 5 μA) but found no differences in input/output responses of field excitatory postsynaptic potentials (fEPSPs) between wild type (WT) and APP mice (Figure 1A). Presynaptic function as determined by paired-pulse facilitation (PPF) was also not altered in APP mice (Figure 1B). We compared LTP induced by theta-burst stimulation (TBS) and revealed that it was significantly lower in APP mice compared to WT (Figures 1C,D). These results suggest that, similar to dorsal hippocampus (Zhang et al., 2021a,b), LTP at CA1 synapse of ventral hippocampus is also impaired in three-month old APP mice.

### Reduction of hippocampal Rac1 activity improves long-term potentiation in APP/PS1 mice

**FIGURE 2:**
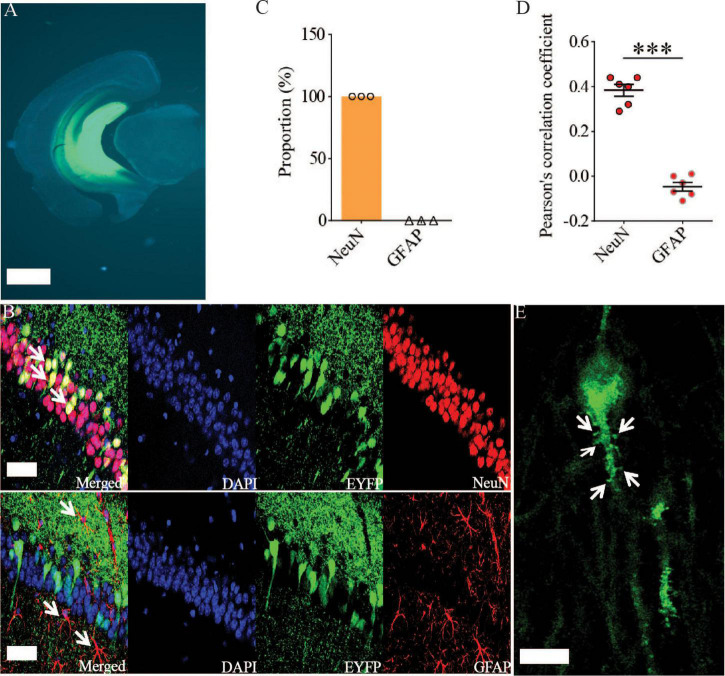
Viral expression of Rac1-N17 in the ventral hippocampus. **(A)** Brain section image showing the expression of Rac1-N17-EYFP in ventral hippocampus. Scale bar: 1 mm. Confocal images **(B)**, summary graph of colocalized cells **(C)**, and Pearson’s correlation coefficient **(D)** showing the expression of Rac1-N17 (EYFP) colocalized with the neuronal marker NeuN, but not with the astrocytic marker GFAP. Arrows indicate neurons and astrocytes. Scale bar: 50 μm. **(E)** Higher magnification confocal image showing expression of Rac1-N17 in the dendrite and spines (arrows). Scale bar: 10 μm. ***P < 0.001.

**FIGURE 3:**
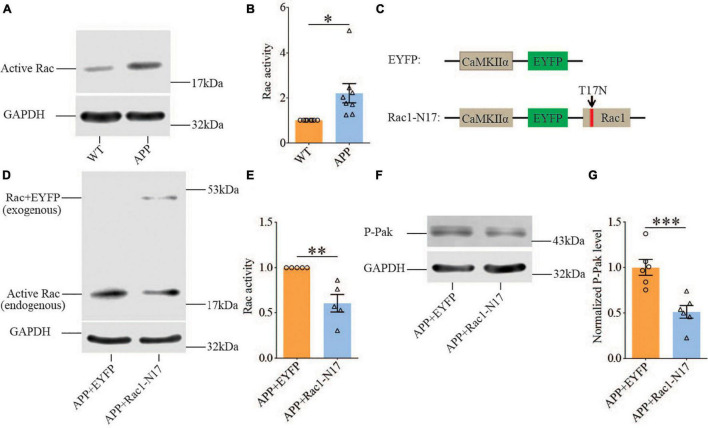
Increased Rac1 activity in APP mice and its reduction by expression of Rac1-N17. Western blots of active Rac1 assay **(A)** and summary graphs **(B)** showing increased level of active form of Rac1 in ventral hippocampus in APP compared to WT mice (WT: *n* = 8 independent experiments from 4 animals for each group, *p* = 0.0127, two-tailed paired *t*-test). AAV virus constructs of control EYFP and Rac1-N17 **(C)**, western blots **(D,F)**, and summary graph **(E,G)** showing decreased level of active Rac1 (*n* = 5 independent experiments from 3 animals for each group, *p* = 0.0335, two-tailed paired *t*-test) and phosphorylated active PAK1/2/3 (P-Pak) (*n* = 6 independent experiments from 3 animals for each group, *p* < 0.001, two-tailed paired *t*-test) in APP mice injected with Rac1-N17 virus (APP + Rac1-N17) compared to those injected with EYFP virus. Endogenous Rac1 refers to Rac1 protein expressed by the endogenous Rac1 gene, while exogenous Rac1 refers to EGFP-Rac1 fusion protein expressed by EYFP-Rac1-N17 viral infections. *P < 0.05, **P < 0.01, and *P* < 0.001.

**FIGURE 4:**
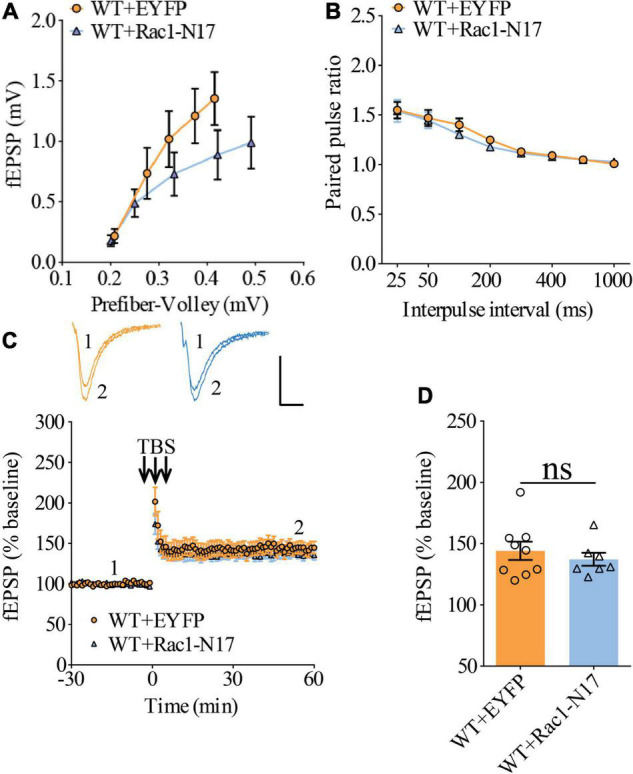
Normal LTP in WT mice overexpressing Rac1-N17 in ventral hippocampus. **(A)** Input–output curves of fEPSP showing no differences between WT mice expressing EYFP and Rac1-N17 (WT + EYFP *n* = 6 slices from 5 mice, WT + Rac1-N17 *n* = 6 slices from 4 mice; genotype: *F*_(1_,_10)_ = 1.187, *p* = 0.301; prefiber volley: *F*_(4_,_40)_ = 35.10, *p* < 0.001; repeated two-way ANOVA). **(B)** Paired pulse ratio showing no differences between WT mice expressing EYFP and Rac1-N17 (WT + EYFP: *n* = 8 slices from 5 mice, WT + Rac1-N17: *n* = 6 slices from 4 mice; genotype: *F*_(1_,_12)_ = 0.223, *p* = 0.645; inter-pulse interval: *F*_(7_,_84)_ = 57.35, *p* < 0.001; repeated two-way ANOVA). **(C)** TBS induced similar LTP at the CA1 synapse in WT mice expressing EYFP and Rac1-N17. Scale bars: 0.4 mV/10 ms. **(D)** Summary graph of last 10 min of recording showing similar levels of LTP in WT expressing EYFP or Rac1-N17 (WT + EYFP: *n* = 9 slices from 5 mice, WT + Rac1-N17: *n* = 7 slices from 4 mice, *p* = 0.486, two-tailed *t*-test).

**FIGURE 5:**
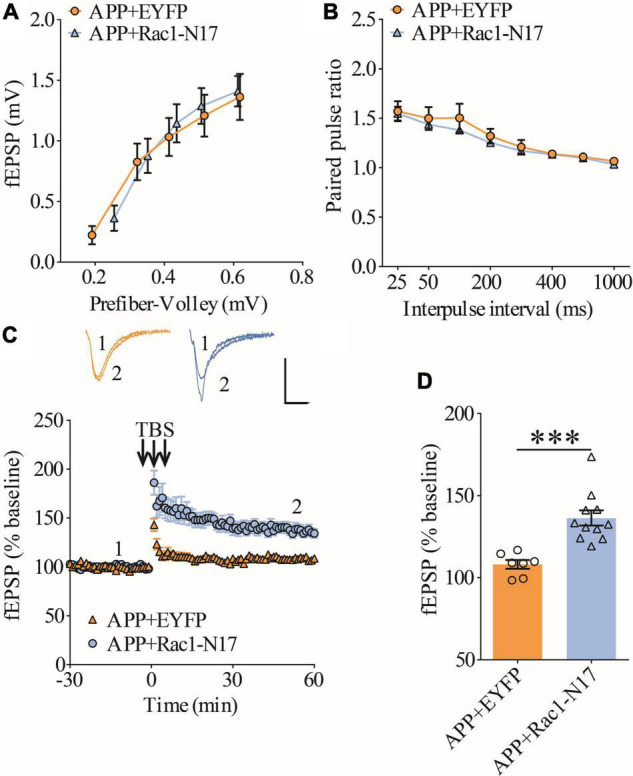
Improved LTP in APP mice expressing Rac1-N17 in ventral hippocampus. **(A)** Input–output curves of fEPSP showing no differences between APP mice expressing EYFP and Rac1-N17 (APP + EYFP: *n* = 8 slices from 4 mice, APP + Rac1-N17: *n* = 7 slices from 4 mice; genotype: *F*_(1_,_15)_ = 0.216, *p* = 0.652; prefiber volley: *F*_(4_,_60)_ = 81.90, *p* < 0.001; repeated two-way ANOVA). **(B)** Paired-pulse ratio analysis showing no differences between APP mice expressing EYFP and Rac1-N17 (APP + EYFP: *n* = 6 slices from 4 mice, APP + Rac1-N17: *n* = 8 slices from 4 mice; genotype: *F*_(1_,_12)_ = 0.472, *p* = 0.505; inter-pulse interval: *F*_(7_,_84)_ = 58.13, *p* < 0.001; repeated two-way ANOVA). **(C)** Enhanced LTP in APP/PS1 mice expressing Rac1-N17 compared to EYFP control. Scale bars: 0.4 mV/10 ms. **(D)** Summary graph of last 10 min of recording showing significantly higher LTP in APP mice expressing Rac1-N17 compared to APP mice expressing EYFP (APP + EYFP: *n* = 7 slices from 4 mice; APP + Rac1-N17: *n* = 11 slices from 4 mice; *p* < 0.001, two-tailed *t*-test). ***P < 0.001.

Previous studies have shown that Rac1 activity is upregulated in AD mice model (Borin et al., 2018; Wu et al., 2019). To investigate whether increased Rac1 activity in the hippocampus is responsible for the synaptic deficits in APP mice, we locally injected AAV virus which expressed a DN Rac1 mutant, Rac1-N17 (the amino acid Threonine at position 17 mutated to Asparagine) fused with EYFP or control EYFP under control of the excitatory neuronal promoter CaMKIIα, bilaterally into the hippocampus. We reasoned that overexpression of DN Rac1 mutant would reduce endogenous Rac1 activity. Immunostaining experiments following the viral injection showed that expression of Rac1-N17 was restricted to ventral hippocampus (Figure 2A). Colocalization of EYFP with neuronal marker, NeuN and the absence of colocalization with astrocytic marker, GFAP confirmed the neuronal and spine expression of Rac1-N17 (Figures 2B–E). Next, we analyzed Rac1 activity in protein lysates prepared from the hippocampus using a Rac1-activation assay. Consistent with the previous study (Wu et al., 2019), the level of active Rac1 was significantly increased in APP mice compared to WT mice (Figure 3A). As expected, the level of active Rac1 was significantly reduced in APP mice expressing Rac1-N17 compared to EYFP control virus (Figures 3C–E). The level of phosphorylated active PAKs (P-Pak) was also reduced in APP mice expressing Rac1-N17 compared to EYFP control virus (Figures 3F,G). The levels of total or phosphorylated LIMK1 and cofilin were not affected in these mice. Electrophysiological recordings showed that expression of Rac1-EYFP had no effect on basal synaptic transmission, PPF, or TBS-LTP in WT mice (Figure 4), but significantly enhanced TBS-LTP without affecting basal synaptic strength or PPF (Figure 5) in APP mice. The expression of control EYFP had no effect on either basal synaptic transmission or LTP in both WT and APP/PS1 mice (Figures 4, 5). These results indicate that reducing Rac1 activity in excitatory hippocampal neurons was sufficient to improve the deficits in TBS-LTP impairment in APP mice.

### Impaired social recognition memory in APP/PS1 mice

**FIGURE 6:**
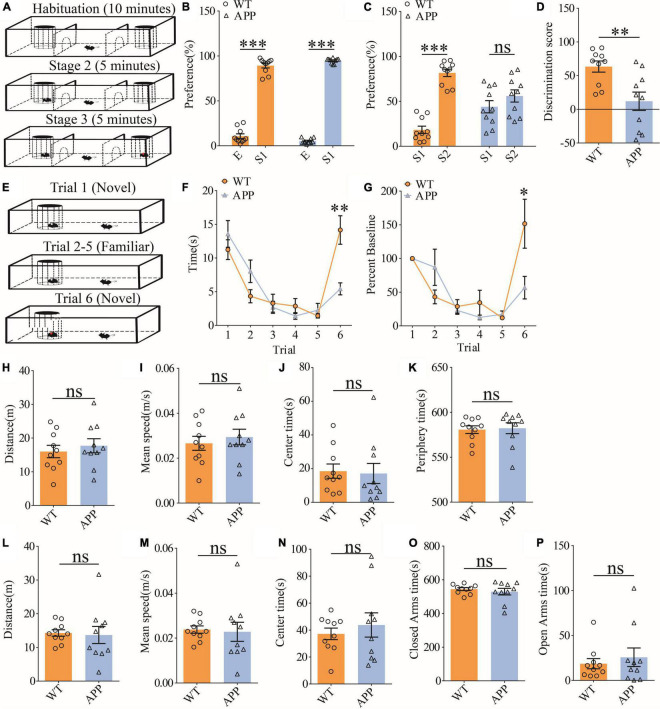
Impaired social recognition memory in APP mice. **(A)** Schematic of the three-chamber social interaction test: stage 1 (habituation), stage 2 (social interaction), and stage 3 (social recognition memory). **(B)** Normal sociability during stage 2 in APP mice (WT: *n* = 10, *p* < 0.001; APP: *n* = 10, *p* < 0.001; two-tailed paired *t*-test). **(C)** Impaired preference for S2 over S1 during stage 3 in APP mice (WT: *n* = 10, *p* < 0.001; APP: *n* = 10, *p* = 0.227; two-tailed paired *t*-test). **(D)** Discrimination scores during stage 3 showing impaired social memory in APP mice (*p* = 0.005, two-tailed *t*-test). **(E)** Schematic of the five-trial social memory test. **(F)** Both WT and APP mice showed gradual habituation for the first stranger during trials 1–5 (WT: *n* = 10, APP: *n* = 10; genotype group: *F*_(1_,_9)_ = 0.016, *p* = 0.901; trial: *F*_(4_,_36)_ = 9.313, *p* < 0.001; repeated two-way ANOVA). On trial 6, APP mice spent significantly less time interacting with the novel stranger compared to WT mice (*p* = 0.002, two-tailed *t*-test). **(G)** Normalized interaction time in the five-trial social test (WT: *n* = 10, APP: *n* = 10; genotype group: *F*_(1_,_9)_ = 0.298, *p* = 0.598; trial: *F*_(4_,_36)_ = 8.517, *p* < 0.001; repeated two-way ANOVA). On trial 6, APP mice showed significantly decreased time interacting with the novel stranger compared to WT mice (*p* = 0.029; two-tailed *t*-test). **(H)** Open field test showing similar travel distance in WT and APP mice (WT: *n* = 10, APP: *n* = 10, *p* = 0.548, two-tailed *t*-test). **(I)** Comparable travel speed between WT and APP mice during open field test (WT *n* = 10, APP *n* = 10, *p* = 0.556, two-tailed *t*-test). **(J)** Comparable time spent in center arena between WT and APP mice during open field test (WT: *n* = 10, APP: *n* = 10, *p* = 0.849, two-tailed *t*-test). **(K)** Comparable time spent in peripheral area between WT and APP mice during open field test (WT: *n* = 10, APP: *n* = 10, *p* = 0.835, two-tailed *t*-test). **(L)** Comparable travel distance between WT and APP mice during elevated plus maze test (WT: *n* = 10, APP: *n* = 10, *p* = 0.830, two-tailed *t*-test). **(M)** Comparable travel speed between WT and APP mice during elevated plus maze test (WT: *n* = 10, APP: *n* = 10, *p* = 0.830, two-tailed *t*-test). **(N)** Comparable time spent in center zone in elevated plus maze test between WT and APP mice during elevated plus maze test (WT: *n* = 10, APP: *n* = 10, *p* = 0.518, two-tailed *t*-test). **(O)** Comparable time spent in closed arms between WT and APP during elevated plus maze test (WT: *n* = 10, APP: *n* = 10, *p* = 0.496, two-tailed *t*-test). **(P)** Comparable time spent in open arms in between WT and APP mice during elevated plus maze test (WT: *n* = 10, APP: *n* = 10, *p* = 0.549, two-tailed *t*-test). *P < 0.05, **P < 0.01, and ***P < 0.001.

To evaluate social interaction and memory in APP mice, we used the three-chamber social interaction test and the five-trial social memory test. The three-chamber social interaction test (Figure 6A) consisted of three stages (stage 1: habituation; stage 2: sociability; and stage 3: social memory). Both APP and WT mice interacted more with stranger 1 (S1) than the empty cage, suggesting that sociability was intact in APP mice (Figure 6B). However, during the social memory stage, WT mice spent more time interacting with the novel stranger (S2) than S1, whereas APP mice interacted equally with S1 and S2, suggesting impaired social recognition memory in APP mice (Figures 6C,D). In the five-trial social memory test (Figure 6E), both WT and APP mice progressively spent less time interacting with the stranger mouse during the repeated exposures (trials 1–5), but showed a significant increase in interaction time when a novel stranger mouse was introduced on trial 6 (Figures 6F,G). However, APP mice spent significantly less time interacting with the novel stranger compared to WT mice on trial 6 (Figures 6F,G), suggesting impaired social recognition memory in APP mice. Collectively, these results suggest that APP mice are deficient in social recognition memory. In the open field test, there were no significant differences between WT and APP mice in travel distance/speed or the amount of time spent in center/periphery zone of the arena (Figures 6H–K). Similarly, there were no differences in total travel distance and the amount of time spent in the closed or open arms during the elevated plus maze test (Figures 6L–P). These results suggest that locomotor activity and anxiety-like behavior were not significantly altered in three-month-old APP mice.

### Reduction of hippocampal Rac1 activity improves social recognition memory in APP/PS1 mice

**FIGURE 7:**
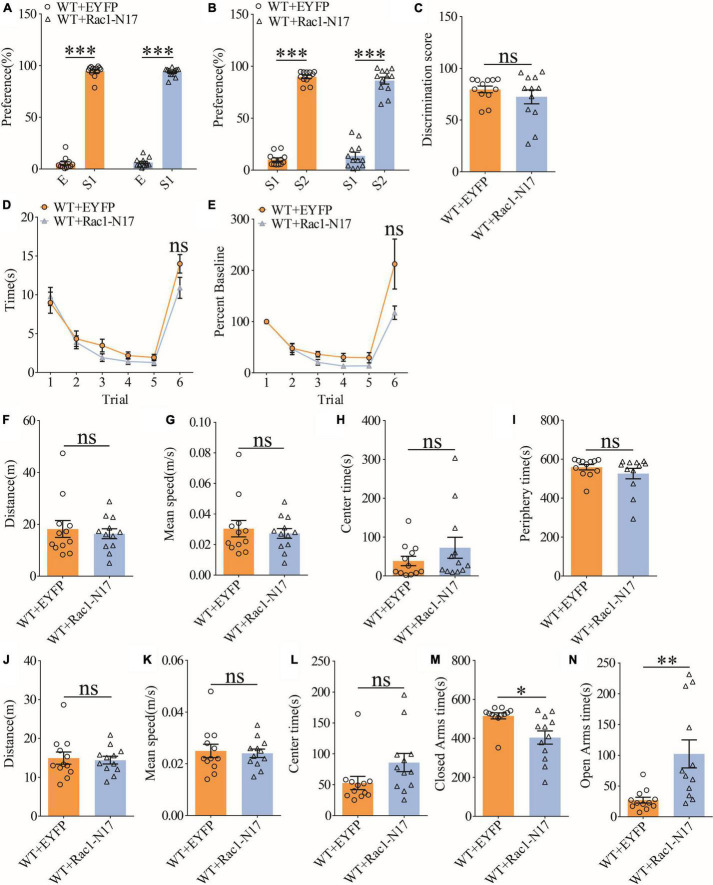
Normal social recognition memory in WT mice overexpressing Rac1-N17. **(A)** Normal sociability during stage 2 of the three-chamber social test in WT mice expressing EYFP or Rac1-N17 (WT + EYFP: *n* = 12, *p* < 0.001; WT + Rac1-N17: *n* = 12, *p* < 0.001; two-tailed paired *t*-test). **(B)** Preference for S2 over S1 during stage 3 of the three-chamber social test in WT mice expressing EYFP or Rac1-N17 (WT + EYFP: *n* = 12, *p* < 0.001; WT + Rac1-N17: *n* = 12, *p* < 0.001; two-tailed paired *t*-test). **(C)** Discrimination scores during stage 3 of three-chamber social test showing no difference in social memory between WT mice expressing EYFP and Rac1-N17 (*p* = 0.345, two-tailed *t*-test). **(D)** Similar performance in the five-trial social memory assay in WT mice expressing EYFP and Rac1-N17 during trials 1–5 (WT + EYFP: *n* = 12, WT + Rac1-N17: *n* = 12; genotype: *F*_(1_,_11)_ = 0.010, *p* = 0.921; trial: *F*_(4_,_44)_ = 11.380, *p* < 0.001; repeated two-way ANOVA) and on trial 6 (*p* = 0.100, two-tailed *t*-test). **(E)** Normalized interaction time showing similar performance in WT mice expressing EYFP and Rac1-N17 during trials 1–5 (WT + EYFP: *n* = 12, WT + Rac1-N17: *n* = 12; genotype: *F*_(1_,_11)_ = 0.532, *p* = 0.485; trial: *F*_(4_,_44)_ = 29.120, *p* < 0.001; repeated two-way ANOVA for trials 1–5) and on trial 6 of the five-trial social test (*p* = 0.083, two-tailed *t*-test). **(F)** Open field test showing comparable travel distance between WT mice expressing EYFP or Rac1-N17 (WT + EYFP: *n* = 12, WT + Rac1-N17: *n* = 12, *p* = 0.635, two-tailed *t*-test). **(G)** Comparable travel speed between WT mice expressing EYFP or Rac1-N17 during open field test (WT + EYFP: *n* = 12, WT + Rac1-N17: *n* = 12, *p* = 0.619, two-tailed *t*-test). **(H)** Comparable time spent in center arena between WT mice expressing EYFP or Rac1-N17 during open field test (WT + EYFP: *n* = 12, WT + Rac1-N17: *n* = 12, *p* = 0.262, two-tailed *t*-test). **(I)** Comparable time spent in peripheral arena WT mice expressing EYFP or Rac1-N17 during open field test (WT + EYFP: *n* = 12, WT + Rac1-N17: *n* = 7, *p* = 0.292, two-tailed *t*-test). **(J)** Comparable travel distance between WT mice expressing EYFP or Rac1-N17 during elevated plus maze test (WT + EYFP: *n* = 12, WT + Rac1-N17: *n* = 12, *p* = 0.784, two-tailed *t*-test). **(K)** Comparable travel speed between WT + EYFP and WT + Rac1-N17 mice during elevated plus maze test (WT + EYFP: *n* = 12, WT + Rac1-N17: *n* = 12, *p* = 0.768, two-tailed *t*-test). **(L)** Comparable time spent in center zone between WT mice expressing EYFP or Rac1-N17 during elevated plus maze test (WT + EYFP: *n* = 12, WT + Rac1-N17: *n* = 12, *p* = 0.080, two-tailed *t*-test). **(M)** Reduced time in closed arms in WT mice expressing Rac1-N17 compared to those expressing EYFP during elevated plus maze test (WT + EYFP: *n* = 12, WT + Rac1-N17: *n* = 12, *p* = 0.010, two-tailed *t*-test). **(N)** Increased time in open arms in WT mice expressing Rac1-N17 compared to those expressing EYFP during elevated plus maze test (WT + EYFP: *n* = 12, WT + Rac1-N17: *n* = 12, *p* = 0.007, two-tailed *t*-test). *P < 0.05, **P < 0.01, and *P* < 0.001.

**FIGURE 8:**
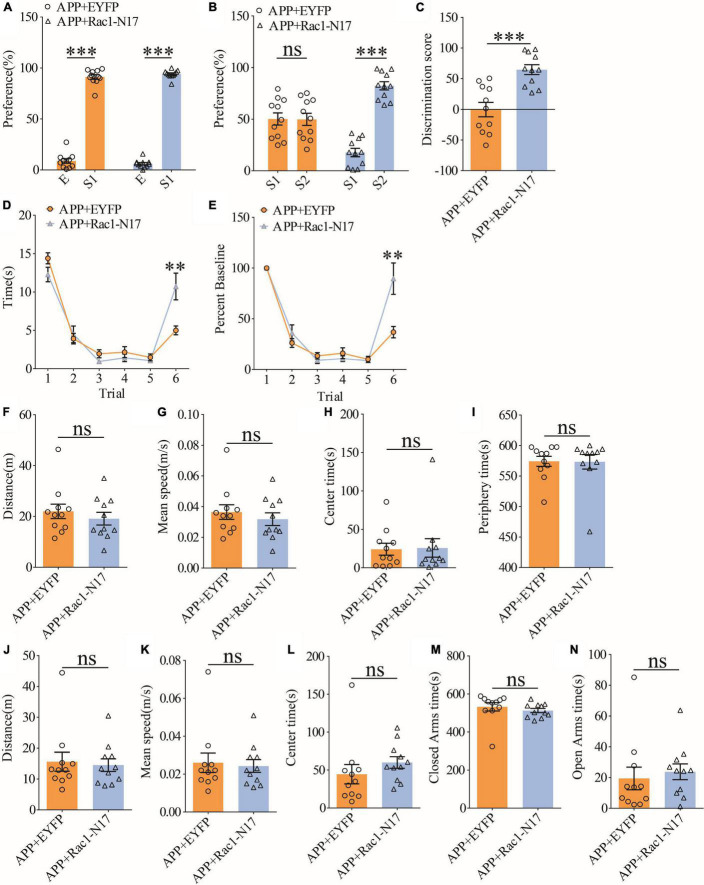
Improved social memory in APP mice expressing Rac1-N17. **(A)** Normal sociability during stage 2 of the three-chamber social test in APP mice expressing EYFP or Rac1-N17 (APP + EYFP: *n* = 11, *p* < 0.001; APP + Rac1-N17: *n* = 11, *p* < 0.001; two-tailed paired *t*-test). **(B)** Preference for S2 over S1 during stage 3 of three-chamber social test in APP mice expressing Rac1-N17 but not in APP mice expressing EYFP (APP + EYFP: *n* = 11, *p* = 0.965; APP + Rac1-N17: *n* = 11, *p* < 0.001; two-tailed paired *t*-test). **(C)** Discrimination scores during stage 3 of the three-chamber social test showing significantly improved social memory in APP mice expressing Rac1-N17 compared to APP mice expressing EYFP (*p* < 0.001, two-tailed *t*-test). **(D)** Similar performance in APP mice expressing EYFP and APP + Rac1-N17 during trials 1–5 of the five-trial social memory assay (APP + EYFP: *n* = 11, APP + Rac1-N17: *n* = 11; genotype: *F*_(1_,_10)_ = 4.172, *p* = 0.068; trial: *F*_(4_,_40)_ = 45.451, *p* < 0.001; repeated two-way ANOVA), but on trial 6, APP + Rac1-N17 mice spent significantly more time interacting with the novel stranger compared to APP + EYFP mice (*p* = 0.009, two-tailed *t*-test). **(E)** Normalized interaction time of the five-trial test during trials 1–5 (APP + EYFP: *n* = 11, APP + Rac1-N17: *n* = 11; genotype: *F*_(1_,_10)_ = 0.628, *p* = 0.446; trial: *F*_(4_,_40)_ = 72.909, *p* < 0.001; repeated two-way ANOVA), and on trial 6 (*p* = 0.007; two-tailed *t*-test). **(F)** Comparable travel distance between APP mice expressing EYFP or Rac1-N17 during open field test (APP + EYFP: *n* = 11, APP + Rac1-N17: *n* = 11, *p* = 0.457, two-tailed *t*-test). **(G)** Comparable travel speed between APP mice expressing EYFP or Rac1-N17 during open field test (APP + EYFP: *n* = 11, APP + Rac1-N17: *n* = 11, *p* = 1.000, two-tailed *t*-test). **(H)** Comparable time spent in center arena between APP mice expressing EYFP or Rac1-N17 during open field test (APP + EYFP: *n* = 11, APP + Rac1-N17: *n* = 11, *p* = 0.910, two-tailed *t*-test). **(I)** Comparable time spent in peripheral arena between APP mice expressing EYFP or Rac1-N17 during open field test (APP + EYFP: *n* = 11, APP + Rac1-N17: *n* = 11, *p* = 0.960, two-tailed *t*-test). **(J)** Comparable travel distance between APP mice expressing EYFP or Rac1-N17 during elevated plus maze test (APP + EYFP: *n* = 11, APP + Rac1-N17: *n* = 11, *p* = 0.767, two-tailed *t*-test). **(K)** Comparable travel speed between APP mice expressing EYFP or Rac1-N17 during elevated plus maze test (APP + EYFP: *n* = 11, APP + Rac1-N17: *n* = 11, *p* = 0.782, two-tailed *t*-test). **(L)** Comparable time spent in center zone between APP mice expressing EYFP or Rac1-N17 during elevated plus maze test (APP + EYFP: *n* = 11, APP + Rac1-N17: *n* = 11, *p* = 0.311, two-tailed *t*-test). **(M)** Comparable time spent in closed arms between APP mice expressing EYFP or Rac1-N17 during elevated plus maze test (APP + EYFP: *n* = 11, APP + Rac1-N17: *n* = 11, *p* = 0.436, two-tailed *t*-test). **(N)** Comparable time spent in open arms between APP mice expressing EYFP or Rac1-N17 during elevated plus maze test (APP + EYFP: *n* = 11, APP + Rac1-N17: *n* = 11, *p* = 0.635; two-tailed *t*-test). **P < 0.01 and ***P < 0.001.

To investigate the functional consequence of reducing Rac1 activity in ventral excitatory hippocampal neurons, we examined social interaction and memory in both WT and APP mice expressing EYFP or Rac1-N17. Expression of Rac1-N17 in WT mice had no effect on social recognition memory in the three-chamber (Figures 7A–C) or the five-trial test (Figures 7D,E), or locomotor activity in the open field test (Figures 7F–I), but reduced anxiety-like behavior in the elevated plus maze in WT mice (Figures 7J–N). These results indicate that reducing Rac1 activity in hippocampal neurons had no effect on social behavior in WT mice. On the other hand, expression of Rac1-N17 in APP mice significantly improved social recognition memory in both three-chamber and five-trial repeated exposure tests (Figures 8A–E), without affecting locomotor activity or anxiety-like behavior (Figures 8F–N). These results suggest that overactivation of Rac1 activity in hippocampal neurons contributes to social memory deficits in APP mice.

## Discussion

Rac1 is a crucial protein involved with learning and memory and its hyperactivity is associated with memory impairments through enhanced forgetting (Shuai et al., 2010; Jiang et al., 2016; Liu et al., 2016, 2018; Lv et al., 2019; Wu et al., 2019). In this study, we tested whether such Rac1-dependent forgetting mechanism would contribute to the social memory impairment observed in a mouse AD model. We showed that Rac1 activity in the hippocampus of APP mice is significantly elevated (Figures 3A,B) in accordance with previous studies (Borin et al., 2018; Wu et al., 2019). This increased Rac1 activity is likely to contribute to the social memory deficit observed in APP mice since the expression of Rac1-N17 in the ventral hippocampus improves impaired social behavior in these mice (Figures 8A–E). The contribution of Rac1 on the social deficit in APP mice is also supported by the recording data showing that the reduced LTP in the ventral hippocampal CA3-CA1 synapse in APP mice is elevated by the expression of Rac1-N17 to reduce Rac1 activity (Figures 5C,D). Therefore, our results reveal that the Rac1-dependent mechanism is an important contributor to the social memory deficit in AD.

The role of Rac1 in learning and memory has been well studied and shown to be distinct in different brain regions. For example, in the amygdala, Rac1 is involved in the auditory fear memory during memory acquisition, consolidation, and reconsolidation (Wu et al., 2014; Gao et al., 2015; Das et al., 2017). Although early studies suggest that Rac1 participates in the acquisition and extinction of hippocampus-mediated memory (Martinez et al., 2007; Sananbenesi et al., 2007; Haditsch et al., 2009), more recent studies emphasize the role of Rac1 in memory forgetting but not in other processes in the hippocampus. The Rac1-dependent forgetting mechanism is initially demonstrated in *Drosophila*, in which the downregulation and upregulation of Rac1 activity is shown to delay and promote the aversive olfactory memory decay, respectively, without affecting its acquisition (Shuai et al., 2010). Such a Rac1-dependent forgetting mechanism has also been shown to affect various hippocampus-mediated memories in mice, including novel object recognition memory, contextual fear memory, spatial memory, and social memory (Jiang et al., 2016; Liu et al., 2016, 2018; Wu et al., 2019). In normal animals, Rac1 activity is elevated for a few days following induction of learning training, and this is accompanied by the extent of memory decay. Manipulating Rac1 activity within this time window by overactivation or inhibition hastens and slows down memory decay, respectively (Lv et al., 2019), suggesting that Rac1 activity is a key determinant of memory forgetting. Therefore, the activity of Rac1 is thought to be tightly regulated within a particular range to maintain animal’s normal behavior. However, the activity of Rac1 can be altered under certain aversive conditions, such as during social isolation. For example, in mice that experience acute social isolation, there is significant elevation of Rac1 activity in the hippocampus, resulting in acceleration of social recognition memory decay without affecting its formation (Liu et al., 2018). Recently, it has been shown that there is a consistent increase in the level of active Rac1 in AD human patients and animal models (Wu et al., 2019). Such elevation of Rac1 in the hippocampus of AD mice is thought to underlie the spatial memory deficit assessed by the Morris water maze test, because specific inhibition of Rac1 activity in the dorsal hippocampus is sufficient to reverse this deficit. However, whether increased Rac1 activity is also responsible for other forms of memory deficits remain unclear, but the results from the present study provide an important step forward by showing that the increased Rac1 activity is also involved in social memory deficits in AD. In addition, the present study demonstrates that inhibiting Rac1 activity in ventral hippocampus is sufficient to improve social memory in APP mice, suggesting that Rac1 alterations in this brain region may be of particular importance. These results are consistent with a previous study showing that ventral hippocampal CA1 is required for social memory formation (Okuyama et al., 2016). It is important to note that other brain regions, such as dorsal CA2, are also involved in social memory (Hitti and Siegelbaum, 2014; reviewed in Tzakis and Holahan, 2019). Consistent with this, our previous study (Zhang et al., 2021a) shows that the manipulation of LIM-domain kinase (LIMK) activity in the dorsal hippocampus can also improve social memory deficit in APP mice. Therefore, both dorsal and ventral hippocampi contribute to social impairments in AD. It would be interesting to further study whether and how these two regions interact.

**FIGURE 9:**
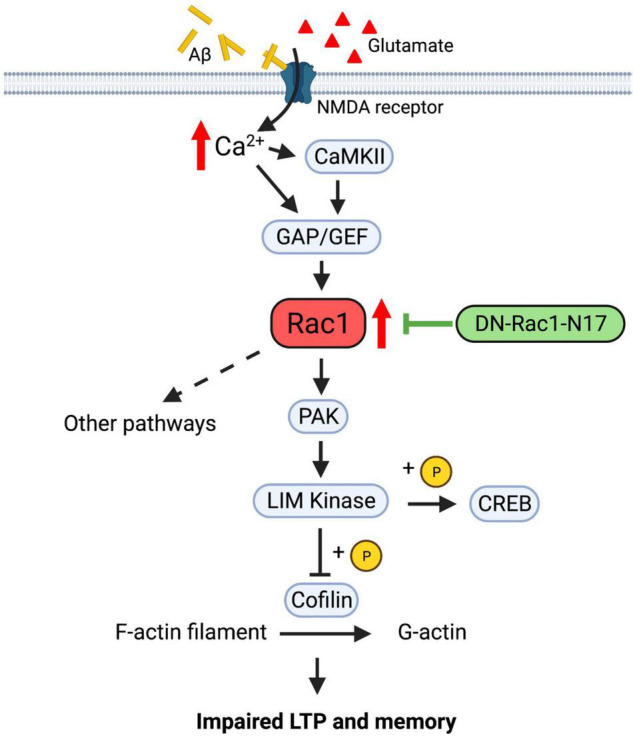
Regulation of LTP and memory by Rac1 in APP mice. In normal animals, Ca^2+^ influx from NMDA receptors activates Rac1 and multiple downstream signaling processes, including PAK-LIMK-cofilin pathway, to modulate actin reorganization, AMPA receptor trafficking and LTP expression. In APP mice, accumulation of Aβ peptides may lead to abnormal activation of NMDA receptors and hyperactive Rac1, which causes dysregulations of downstream proteins, including PAK, LIMK and cofilin, to impair LTP and memory. Expression of Rac1-N17 in hippocampal neurons reduces Rac1 activity and restores the function of some of its downstream proteins such as PAKs, thus improving LTP and memory in APP mice.

Despite that Rac1 plays a role in learning and memory, the underlying signaling pathway is not clear. Rac1, as a member of the Rho family of small GTPases, can potentially contribute to the regulation of memory through the modulation of the actin cytoskeleton and synaptic plasticity (Figure 9). Since the activation and translocation of Rac1 in the hippocampus during training can be blocked by the infusion of NMDA receptor inhibitors (Martinez et al., 2007), NMDAR receptors may be a crucial upstream mediator of Rac1 activation. Signals from NMDA receptors could be relayed by guanine nucleotide-exchange factors (GEFs) and GTPase-activating proteins (GAPs), upstream regulators of Rac1 that activate and inactivate Rac1, respectively (Rossman et al., 2005; reviewed in Bos et al., 2007; Cromm et al., 2015). Alterations of GEF and GAP activity are associated with impaired synaptic plasticity and cognitive deficits (Cahill et al., 2009; Kasri et al., 2009; Oh et al., 2010; Clement et al., 2012; Berryer et al., 2013; Zamboni et al., 2016). Rac1 can activate multiple downstream pathways to mediate cytoskeletal remodeling. One of these pathways involves the activation of P21-activated kinases (PAKs), which in turn activates LIMKs, leading to phosphorylation and inactivation of the actin regulator cofilin (Figure 9). Disruptions in PAK-LIMK-cofilin signaling are associated with impairments in synaptic function and memory (Meng et al., 2002, 2004, 2005; Asrar et al., 2009; Zhou et al., 2009; Huang et al., 2011). Other pathways include the WASP-family verprolin-homologous protein (WAVE) complex which can be recruited by active Rac1 to promote actin polymerization and branching *via* the activation of mDia and Arp2/3 complexes (Eden et al., 2002; reviewed in Faix and Grosse, 2006; Chen et al., 2017; Gao et al., 2019), both being reported to regulate memory forgetting (Gao et al., 2019). In addition to the actin cytoskeleton, Rac1 may also exert its effects on synaptic function *via* actin-independent pathways. For example, a recent study reported that Rac1 can affect PKCλ and PKMζ kinases (Cui et al., 2021). Both PKCλ and PKMζ are critically involved in AMPAR surface expression, and LTP induction and maintenance (reviewed in Sacktor, 2011; Ren et al., 2013). Furthermore, Rac1 may regulate LTP *via* gene transcription related processes. It has been reported that Rac1 is involved in the JNK signaling pathway (Kukekov et al., 2006), which is required for hippocampal LTP (Seo et al., 2012). In addition to cofilin, LIMK1 can directly phosphorylate and activate cAMP-responsive element-binding protein (CREB) to promote gene transcriptions and LTP maintenance (Yang et al., 2004; Todorovski et al., 2015). Because Aβ peptides can directly stimulate NMDA receptors (Texidó et al., 2011), it is possible that abnormal activation of NMDA receptors by Aβ peptides leads to hyperactivation of Rac1, resulting in alterations in any of the aforementioned signaling processes to cause LTP and social memory deficits in APP mice. Our data that the level of P-PAKs (P-PAK1/2/3) is reduced in APP mice expressing Rac1-N17 suggest that changes in the PAK-LIMK-cofilin pathway may represent an important mechanism by which overactivation of Rac1 impairs LTP and memory in APP mice. These results are consistent with previous studies showing that manipulations of LIMK1 can improve social memory deficit in APP mice (Heredia et al., 2006; Henderson et al., 2016, 2019; Leung et al., 2018; Gory-Fauré et al., 2021; Zhang et al., 2021a). Although we found no differences in the protein level and activity of LIMK1 and cofilin in APP mice expressing Rac1-N17 using total hippocampal protein lysates, we cannot rule out the possibility that synaptic levels of these proteins are affected by the expression Rac1-N17.

In summary, this study shows that increased Rac1 activity contributes to impaired LTP in ventral hippocampus and social recognition memory. Future experiments would be to address how the Rac1 activity is enhanced by examining its upstream GEF and GAP in AD models and human patients. Defining the details of Rac1 signaling processes may provide potential new therapeutic strategies and molecular targets to treat AD and related brain disorders.

## Materials and methods

### Housing, maintenance, and use of mice

APP/PS1 transgenic mice (#34829-JAX) on mixed C57BL/6;C3H genetic background were obtained from the Jackson Laboratory. The mice were inbred and housed (2–5 mice per cage) on a 12/12 h light/dark cycle with food and water *ad libitum*. The following PCR primers were used for genotyping APP mice: oIMR 1644: AATAGAGAACGGCAGGAGCA; oIMR 1645: GCCATGAGGGCACTAATCAT. All experimental procedures were conducted according to the guidelines of the Canadian Council on Animal Care (CCAC) and approved by the Animal Care Committee at the Hospital for Sick Children, Canada. All experiments were performed blind to the genotype of the mice. Both male and female mice were used but no differences were noted between sexes, therefore the data were pooled together for statistical analyses between genotypes. The age of the mice ranged from 3 to 4 months.

### Surgical procedures

For viral injections, the AAV2/DJ-CaMKIIα-Rac1-N17 (Rac1 fused to EYFP, 7.6 × 10^12^) and AAV2/DJ-CaMKIIα-EYFP (1.3 × 10^13^) (produced through Canadian Neurophotonics Platform, Laval University, Canada) were injected bilaterally to the ventral hippocampus. Briefly, mice were anesthetized with isoflurane (2.0–2.5% in 1 L/min oxygen) and placed onto a stereotaxic frame. Body temperature was maintained at 37°C using a temperature controller. A midline scalp incision was made followed by craniotomies using a 0.6 mm drill bit. The virus was injected by a microsyringe pump with the injection speed of 0.3 μL/min. Six minutes after the injection, the needle was retrieved slowly. Injection site of ventral hippocampus (AP: −3.16 mm, DV: −4.00 mm, and ML: ±3.20 mm). At the end of the surgery, the skin was sutured sequentially, and the animals were given hydration and painkillers. The surgically operated mice were recovered for 3–4 weeks to allow for Rac1-N17 and EYFP expression before behavior tests were performed. The expression pattern of Rac1-N17 and EYFP as well as the injection sites were confirmed by immunohistochemical staining of fixed brain sections after behavior tests.

### Slice electrophysiology

All electrophysiological recordings were done at the Schaffer collateral-commissural pathway in ventral hippocampus as previously described. In brief, the mouse brains were removed and 350 μm brain slices prepared in ice-cold artificial cerebrospinal fluid (ACSF) saturated with 95 O_2_/5% CO_2_. ACSF contained (in mM): 120.0 NaCl, 3.0 KCl, 1.2 MgSO_4_, 1.0 NaH_2_PO_4_, 26.0 NaHCO_3_, 2.0 CaCl_2_, and 11.0 D-glucose. The slices were recovered at 28°C for at least 2 h before a single slice was transferred to a submersion chamber constantly perfused with 95% O_2_/5% CO_2_ saturated ACSF. Perfusion flow rate was maintained constant at 2 ml/min. Synaptic transmission was evoked by stimulation at 0.067 Hz and recorded with glass pipettes (3–4 MΩ) filed with ACSF. For input-output field potential experiments, the stimulus intensity was increased gradually (0, 1, 2, 3, 4, and 5 μA). PPFs were obtained at inter-pulse intervals of 25, 50, 100, 200, 300, 400, 500, or 1,000 ms, and calculated as the ratios of the second response peak values over the first response peak values. LTP was induced by three trains of theta burst stimulations (TBS, five pulses at 100 Hz every 200 ms) with an intertrain interval of 10 s. LTP was calculated and statistically evaluated by comparing the mean values of the last 10 min of the recording and the mean values of the entire baseline. All data acquisition and analysis were done using pCLAMP 10.7 (Axon Instruments, Foster City, CA, United States).

### Active Rac1 assay

Mouse hippocampus was harvested and snap freeze in dry ice before being homogenized in cold lysis buffer which contains (in mM): 20 Tris–HCl (pH 7.5), 150 NaCl, 1 EDTA, 1 EGTA, 1% Triton X-100, 2.5 sodium pyrophosphate, 1 β-glycerophosphate, 1 Na3VO4, 20 NaF, and 1% protease inhibitor cocktail and phosphatase inhibitor. After shaking for one hour in 4°C, insoluble debris were removed *via* centrifugation at 10,000 rpm (for 15 min, at 4°C). The protein concentration was measured *via* BCA assay (Thermo-Fisher, #23225). One milliliter protein lysate with a concentration of 500 μg/ml was mixed with 10 μg GST-tagged PAK-PBD agarose beads (Cytoskeleton, PAK02) as instructed by the company protocol. After incubation in 4°C overnight, beads were washed for three times with cold lysis buffer before being separated on SDS-PAGE (15%) gels. Proteins were then transferred onto nitrocellulose membranes, blocked by 5% skim milk and incubated with primary antibody against total Rac1 (CST, #2465), P-PAK1 (CST, #2605S), and GAPDH (CST, #2118S) overnight at 4°C. The membrane was then incubated with HRP-conjugated goat anti-Rabbit IgG (CST, #7074S) for 1 h at room temperature. Then the blot was washed and developed using an enhanced chemiluminescence (Thermo-Fisher, #34579) method of detection and analyzed by Image Studio Lite software (Licor) as per manufacture’s instruction.

### Histology and immunohistochemistry

For immunohistochemistry staining, mice were anesthetized with injected ketamine (15 g/ml) followed by transcardial perfusion with 50 mL of pre-cooled 1X PBS sequentially and 4% paraformaldehyde 50 ml (4% PFA). The brain is taken and post-fixed in 4% PFA solution at 4°C overnight. The next day, after being thoroughly washed with 1X PBS, brain was embedded in 4% agarose gel. The brain was sliced to 40 μm coronal sections by a vibratome at room temperature. Sections were washed with PBS, incubated in blocking solution (0.3% Triton, 5% BSA in 1X PBS) for 1 h, with primary antibodies (for NeuN: 1:1000, CST, #12943; for GFAP: 1:500, CST, #3670) overnight at 4°C. The slides were then washed with PBS and incubated with secondary antibodies (dissolved in 0.05% Triton, 5% BSA in 1X PBS with dilution of 1:1,000; AlexaFluor 555, Thermo-Fisher, #A32794 and #A32773) at room temperature for 4 h. Following washing, the coverslips were mounted using Antifade mounting medium with DAPI (MJS Biolynx, #VECTH180010) for image collection. Images were collected using a Leica epi-fluorescence microscope and a Nikon A1R or Leica SP8 lightning confocal microscope, under a 10× and 60× objective, respectively. The excitation used were 402 nm for DAPI, 488 nm for GFP, and 562 nm for Red. The emission used were 460 nm for DAPI, 509 nm for GFP, and 580 nm for Red. For colocalization analysis, Pearson’s correlation coefficients were calculated for images using Coloc2 plugin from ImageJ. For each group, 4–6 images were analyzed from each of 6 mice. GFP was displayed as channel A and anti-NeuN or anti-GFAP were displayed as channel B. Channel thresholds were set as to include the full range of data as displayed in the colocalization tool 2D histogram. Average Pearson’s coefficients for each mouse were plotted using Prism software and statistical analysis was done using Mann–Whitney test.

### Behavioral tests

Animals were tested at the age of 3 ± 0.5 months. All behavioral tests were performed during the light cycle. The mice were tested in open field, elevated plus maze, three-chamber social interaction and five-trial repeated social test. At least 3-day intervals were given after each test. The open field apparatus was a rectangular Plexiglas box (40 cm long × 40 cm wide × 35 cm high) comprising four walls and an open roof. The illumination in the room was provided by centrally placed in-ceiling dim lights. All mice were individually tested in one 5 min session. Each subject was introduced to the apparatus in the same place of the arena near the center and allowed to explore the apparatus for 10 min. The apparatus was cleaned thoroughly with 75% ethanol before each subject was tested. The movement of the mouse was video tracked and analyzed off-line using ANY-maze software (Untied States). The box was divided into central (center 20 cm diameter) and peripheral fields for analysis. The elevated plus maze was composed of two open arms (35 cm long × 5 cm wide) and two closed arms of the same size with 10 cm high walls. The apparatus was placed 50 cm above the ground. The tested mice were individually placed in the center and allow for 10 min free exploration. The entries to and time spent in the open arms, center zone and closed arms were recorded. The maze was cleaned thoroughly with 75% ethanol before each mouse was tested. Traces of movement were tracked and analyzed off-line using ANY-maze software (United States). The movement distance, average speed, the entries to and time spent in the open arms, center zone, and closed arms were recorded. The maze was cleaned thoroughly with 75% ethanol between mice. The movement was tracked and analyzed using ANY-maze software (United States). Three chambers (60 cm long × 40 cm wide × 22 cm high) for social interaction connected by removable partitions in the plexiglass walls, which allowed animals to freely moving between the chambers. Mice were handled twice a day, 3 days before the test. Prior to the day of test, the handled mice were each habituated to the empty apparatus for 5 min. Stranger mice were contained in a cylindrical wired cage (8 cm diameter and 17 cm high) with bars spaced 1 cm apart placed in left and/or right chamber. The middle chamber was left empty all the time. Each test session consisted of three stages: stage 1: 10 min habituation stage with two empty cages; stage 2: 5 min sociability test with an unencountered stranger mouse (S1) and an empty cage; stage 3:5 min social memory test with the previously encountered stranger (S1) and a second novel stranger (S2). Each stage was separated by a 45 s^–1^ min interval. The amount of interaction (i.e., sniff time when the animal oriented its nose within 0.5 cm or physical contact of the mouse contained in the wired cage) was recorded. Data were analyzed as a percentage time spent investigating the target cage over the total time interacting with either cage using ANY-maze software (IL, United States). For five-trial social interaction test, the subject mouse was placed in a chamber (40 cm long × 20 cm wide × 22 cm high) and presented with a same sex juvenile, strange mouse in a cylindrical wired cage (8 cm diameter and 17 cm high) with bars spaced 1 cm apart. Six consecutive 1 min trials with a 30–45 s inter-trial interval was tested for each subject. On the last trial, a novel stranger juvenile mouse of the same sex was presented in the cage. The amount of interaction was recorded as the sniff time when the animal oriented its nose within 1 cm of the stranger mouse in the wired cage. The normalized baseline values were calculated by dividing the amount of interaction in each trial (2–6) to that of trial 1. Data were analyzed using ANY-maze software (IL, United States).

### Statistical analyses

All the averaged data in the graphs were stated as mean ± SEM. and statistically evaluated by Student’s *t*-test for comparisons of two groups or by ANOVA (one-, two-way or repeated measures, as appropriate) for comparisons of more than two groups followed by *post hoc* Fisher s LSD multiple comparison test using the SPSS program. For each set of data, values were first tested for their normal distribution using the Shapiro–Wilk test of normality. Data followed Gaussian normal distribution unless it is stated otherwise. *p* < 0.05 was considered significant and indicated with *p < 0.05, ^**^p < 0.01, ^***^p < 0.001. The details of statistical data, including statistical methods, *p*-values and sample size, were provided in respective figure legends.

## Data availability statement

The original contributions presented in this study are included in the article/supplementary material, further inquiries can be directed to the corresponding authors.

## Ethics statement

All experimental procedures were conducted according to the guidelines of the Canadian Council on Animal Care (CCAC) and approved by the Animal Care Committee at the Hospital for Sick Children, Canada.

## Author contributions

CZ, TX, and ZJ designed the study. HWZ, YB, HRZ, AL, RG, DL, and YM performed the experiments. HWZ, YB, HRZ, AL, DL, CZ, and SL analyzed and interpreted the data. HWZ, YB, HRZ, CZ, TX, and ZJ wrote the manuscript. All authors read and approved the final manuscript.
